# A Case of Eccrine Mucinous Carcinoma Involving Scalp

**DOI:** 10.7759/cureus.16469

**Published:** 2021-07-18

**Authors:** Ramsha Saleem, Sachin Vaidya

**Affiliations:** 1 Dermatology, The Queen Elizabeth Hospital, Adelaide, AUS

**Keywords:** scalp lump, eccrine carcinoma, cutaneous carcinoma, sebaceous cyst, primary cutaneous mucinous carcinoma

## Abstract

The report presents a case of a 67-year-old female with a long-standing lump on the scalp. After its excision the histopathology revealed consistency with eccrine mucinous carcinoma. These neoplasms are quite rare with only around 100 cases reported since 1951. The report concludes the importance of encouraging follow-up of cutaneous lesions among patients as well as ongoing research to better identify and manage the tumor.

## Introduction

Primary cutaneous mucinous carcinoma (PCMC) is a rare neoplasm arising from sweat glands and primarily affecting the head and neck area [1]. It was first described by Lennox et al. in 1951, and till 2018, only around 100 cases were reported [2]. The difficulty of distinguishing this primary neoplasm from metastatic carcinoma of non-cutaneous origins with primary sites in lungs, breasts, or colon presents a diagnostic challenge [3]. Mimicry of this entity by other epithelial lesions and mucin-producing mesenchymal lesions also adds to these challenges [4]. Its clinical characterization is not only asymptomatic, slow growing, and benign appearing but it also has a 30% rate of local recurrence [5]. Apart from this, the clinical presentation of PCMC is known to be non-specific with differential diagnoses including lacrimal sac tumor, sebaceous carcinoma, melanoma, pyogenic granuloma, epidermoid cyst, lipoma, pilomatricoma, neuroma, cystic basal cell carcinoma, and metastatic adenocarcinoma [6,7]. According to Oh and Kim [8], there is controversy surrounding the histogenesis of PCMC. While several studies report the neoplasm to have shown an eccrine differentiation [9,10], several others reported an apocrine origin [11]. For our case, however, we concluded the tumor to be of eccrine differentiation based on histopathology. PCMC’s occurrence is most commonly observed on the eyelid followed by the scalp, face, axilla, abdominal wall, vulva, neck extremity, canthus, groin, and ear [8,10].

We present a rare case of eccrine mucinous carcinoma on the scalp. The scalp is anatomically characterized by its stratified structure ‘(consisting of epidermis, dermis, subcutis, epicranial aponeurosis and the adjacent periosteum and skull)’ as well as the closely occurring adnexa that are surrounded by a network of lymphatics and vessels [12]. Among other tumors, a number of different types of tumors can arise in this region due to its structural characteristics including neoplasms that are predominantly benign.

In our patient, a long-standing lump recently started to increase in size. Based on the initial impression, it was advised to wait and watch; however, on a sixth-month review, the size of the cyst along with its symptoms had increased.

## Case presentation

**Figure 1:**
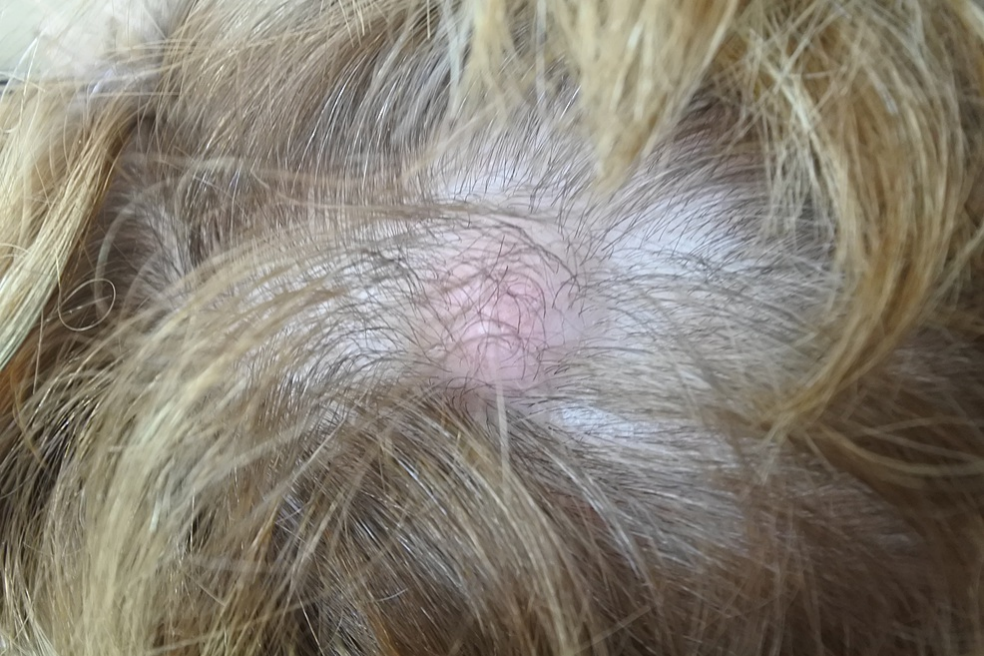
Smooth lump in right occipital region of scalp.

A 67-year old female presented with a smooth, solitary lump on scalp in the right occipital region (Figure 1).

**Figure 2:**
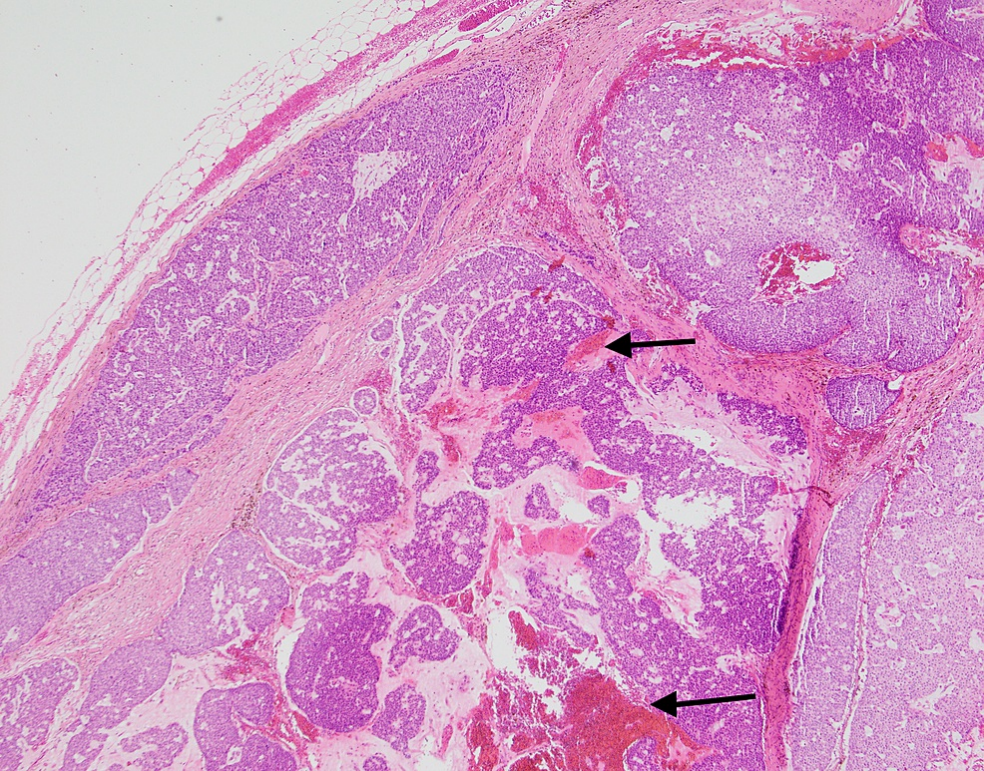
Large, circumscribed, nodular tumor occupying almost full thickness of reticular dermis. Abundant PAS positive mucin in the stroma (arrow). PAS, periodic acid–Schiff.

**Figure 3:**
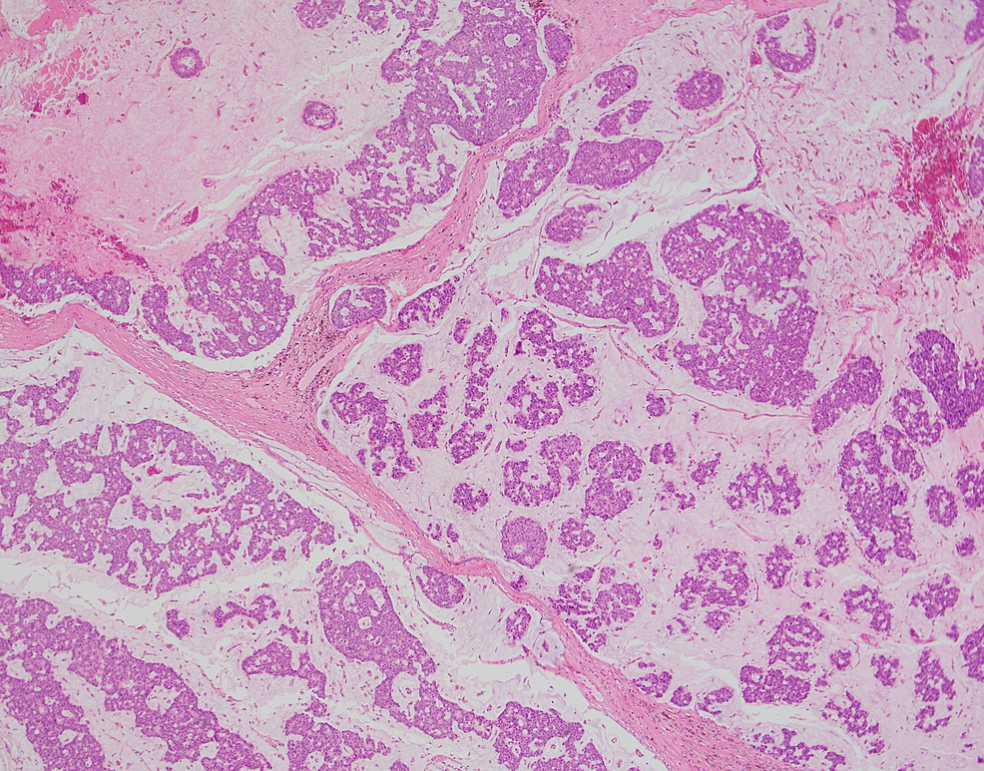
Tumor consisting of multiple masses of neoplastic epithelium with poroid appearance.

On examination, a 3 x 3 cm smooth, firm, non-mobile lump was palpable with overlying scalp hair. The initial impression was that of a sebaceous cyst and she was advised to wait and watch for any changes to the features of the lesion or development of any new symptoms. On review after six months, the size of the lesion increased to 4 x 4 cm and the patient reported progressively worsening itching on the overlying skin. A decision was made to completely excise the cyst. Histopathological analysis from the lesion confirmed a mucinous eccrine carcinoma of skin (Figures 2, 3).

## Discussion

Primary eccrine mucinous carcinoma of the skin is a rare pathological entity. Typically, these present as slow-growing, soft, and painless lumps that have been present for several years [5]. Standard treatment for primary mucinous eccrine carcinoma is surgical removal with 1 cm margin or Moh's micrographic surgery if the expertise is available [7].

With only a few cases reported, a number of different statistics are available regarding the recurrence of PCMC. While one study reported eccrine mucinous carcinoma to have a 19.6% tendency for local recurrence and 6.1% tendency for metastasis after the surgical treatment [3], another reports local recurrence of 29.4% and a metastatic rate of 9.6% [4] and yet another observes a 30% local recurrence and a 2.7% rate of metastases [5]. It is, therefore, critical that data collection and case reporting on PCMC remain ongoing.

Furthermore, the PCMC is mostly reported in middle-aged and older individuals [8] with a 2:1 predominance in men as compared to women [1]. The neoplasm also has an increased predilection to white rather than individuals of African American, Asian, or Indian ethnicities [1].

There is limited neoplasm with predominant sebaceous differentiation, and the variation in the diagnosis of sebaceous lesions as carcinoma even by specialist dermato-pathologists ranges from 5% to 57% [13]. Death following mucinous carcinoma is rare with >5 cases reported [3,4].

## Conclusions

This case highlights the importance of maintaining a high index of suspicion of malignancy in benign-appearing cutaneous lesions, especially if the lesion is evolving or there is onset of new symptoms. Given that the risk of recurrence is primarily associated with incomplete margin excision, it is imperative that complete removal of tumor is achieved even at the cost of re-excision. Considering the risk of recurrence and metastasis, patients should be counselled about the importance of follow-up and detection of regional lymphadenopathy. This case also sheds light on the significance of differentiating primary cutaneous tumors from systemic metastasis.
